# Personal

**Published:** 1895-08

**Authors:** 


### Personal.

Dr. Daniel Lewis, of the city of New York, was elected president of the State board of health at a meeting held in Albany, July 23, 1895. Dr. Lewis is one of the best known medical men in the state, has been president of the medical society of the state of New York and of the medical society of the county of New York, and is now vice-president of the New York academy of medicine. His unanimous election to the honorable and responsible office of president of the State board of health is a matter of supreme satisfaction from one end of the state to the other.

---

Dr. George B. Fowler, of New York, has been appointed health commissioner of that city vice Dr. Cyrus Edson, resigned. Dr.

Fowler was graduated from the New York College of Physicians and surgeons in 1871, and has since been in the active and successful practice of his profession, in which he has taken extremely high rank. He is a member of the County Medical Society and of the Academy of Medicine, and is visiting physician to Bellevue Hospital and the New York Infant Asylum.

---

Dr. James T. Jelks assumed editorial charge of the *Hot Springs Medical Journal*, beginning with the June issue, 1895. Dr. Jelks is professor of gynecology and syphilology in the Barnes Medical College, St. Louis. His ability as a writer and teacher is a guarantee of able editorial conduct of the *Hot Springs Medical Journal.*

---

Dr. Richard H. Satterlee, of Buffalo, has been appointed oculist for the Buffalo street railway company and also for the Buffalo, Rochester & Pittsburg railway. It is a wise provision on the part of the street railway to inspect the vision of its motor men and to insist upon the highest exercise of that function.

---

Dr. I. N. Love, of St. Louis, editor of the *Medical Mirror*, departed for Europe, late in July, to attend the meeting of the British medical association. He will return in season to attend the meeting of the Mississippi Valley medical association in Detroit, September 4–7, 1895.

---

Dr. L. S. McMurtry, of Louisville, surgeon in charge, announces that the Jennie Casseday Infirmary for women will be closed from August 1 to September 5, 1895. During his vacation Dr. McMurtry will visit Buffalo and will spend some time at Niagara Falls.

---

Dr. Charles A. L. Reed, of Cincinnati, has closed his hospital in St. Leger place and sailed for Europe to join his family. He will return with Mrs. Reed and children early in September, when his private hospital will be reopened for patients.

---

Dr. Bransford Lewis, of St. Louis, having resigned his position in the Missouri medical college, has been elected professor of genito-urinary surgery in the College of Physicians and Surgeons and genito-urinary surgeon to the Baptist hospital.

---

Dr. H. Y. Grant, of Buffalo, accompanied by Mrs. Grant, sailed Monday, July 29, 1895, on the North German Lloyd steamer "Havel" for a two months' tour in Europe.

Dr. Frederick W. Smith, of Syracuse, has been appointed a member of the state board of health in place of Dr. F. O'Donohue, whose term of office has expired.

---

Dr. John B. Murphy, of Chicago, sailed for Europe, on the "Etruria," July 20, 1895, to attend the meeting of the British medical association. He will be absent about a month.

---

Dr. A. W. Hurd, superintendent of the Buffalo state hospital, sailed for Europe, July 20, 1895, to be absent a few weeks. He was accompanied by Mrs. Hurd.

---

Dr. Henry J. Mulford, of Buffalo, has removed from 56 Allen street to 466 Franklin street, where he will continue the practice of his specialty, laryngology.

---

Dr. John Hauenstein, of Buffalo, has removed from 499 Washington street to his fine up-town residence, 309 Elmwood avenue.

---

Dr. Walter B. Dorsett, of St. Louis, has removed his office from 3323 Lucas avenue to his residence, 3941 West Bell place.

---

Dr. D. W. Harrington, of Buffalo, is at present enjoying a tour in Europe and is announced to return about September 1, 1895.

---

Dr. Joseph Fowler, of Buffalo, has removed his office from 33 Church street to 141 Delaware avenue.

